# The fellowship of the ring: species-associated effects of fairy ring fungi on soil, microbiota, and vegetation in a managed ornamental grassland

**DOI:** 10.1093/ismeco/ycag177

**Published:** 2026-06-22

**Authors:** Maurizio Zotti, Mohamed Idbella, Giuseppina Iacomino, Astolfo Zoina, Roberta Di Lecce, Alberto Minelli, Maria Antonietta Rao, Giuliano Bonanomi, Stefano Mazzoleni

**Affiliations:** Department of Agricultural Sciences, University of Naples Federico II, Piazza Carlo di Borbone 1, Portici 80055, Italy; College of Agriculture and Environmental Sciences, AgroBioSciences (AgBS) Program, Mohammed VI Polytechnic University, Ben Guerir 43150, Morocco; Department of Agricultural Sciences, University of Naples Federico II, Piazza Carlo di Borbone 1, Portici 80055, Italy; Department of Agricultural Sciences, University of Naples Federico II, Piazza Carlo di Borbone 1, Portici 80055, Italy; Department of Agricultural Sciences, University of Naples Federico II, Piazza Carlo di Borbone 1, Portici 80055, Italy; Department of Agricultural and Food Sciences, Alma Mater Studiorum, University of Bologna; Department of Agricultural Sciences, University of Naples Federico II, Piazza Carlo di Borbone 1, Portici 80055, Italy; Department of Agricultural Sciences, University of Naples Federico II, Piazza Carlo di Borbone 1, Portici 80055, Italy; Task force of Microbiome Study; Department of Agricultural Sciences, University of Naples Federico II, Piazza Carlo di Borbone 1, Portici 80055, Italy; Task force of Microbiome Study

**Keywords:** Basidiomycota, *Marasmius oreades*, *Amanita vittadinii*, *Clitocybe collina*, fungal saprotrophs, self-DNA inhibition, metabarcoding

## Abstract

Fairy ring-forming fungi may alter soil physicochemical properties through mycelial decomposition of organic matter, with potential cascading effects on plants and soil microbiota. This study investigated fairy rings formed by three co-occurring fungal species, *Marasmius oreades, Amanita vittadinii*, and *Clitocybe collina*, in the ornamental grassland of the Royal Palace of Caserta. Along outer, active-front, and inner transect zones, we assessed fungal DNA distribution, vegetation, soil physicochemical properties, and soil microbial communities through metabarcoding. Reads assigned to the dominant fairy ring-forming fungi peaked at the active fungal front and remained detectable in inner soils, consistently with the persistence of fungal residues after mycelial passage and with a model of centrifugal ring development associated with self-DNA accumulation. Across the three species, the active front showed a common functional syndrome, including increased CO₂ flux, soil acidification, and nutrient mobilization. However, the ecological consequences, in terms of impact on biological communities, differed among fungal species and were consistent with distinct decomposition-related strategies. *M. oreades* was associated with high soil hydrophobicity and showed the strongest effects on both vegetation and soil microbial communities, supporting its recognition as the only clear ecosystem engineer in this system. *A. vittadinii* mainly promoted nutrient release and soil biochemical changes, whereas *C. collina* was consistent with lower decomposition intensity and produced weak or negligible effects on the managed ornamental grassland biota. Overall, the co-occurrence of different fairy ring-forming fungi within the same field conditions revealed both shared front-related processes and species-associated differences in their impacts on grassland biota.

## Introduction

Fairy rings are recurrent spatial patterns in grasslands, including both seminatural and managed grasslands [1, 2]. They are formed by the radial expansion of basidiomycete mycelia in soil [3], which can produce alterations in the vegetation often associated with the emergence of sporophores in consistently circular patterns [4–6]. Fairy ring-forming fungi are taxonomically and functionally diverse: in grasslands, they are mainly represented by free-living saprotrophic fungi, including species of *Marasmius, Agaricus, Calocybe, Lepista, Lycoperdon*, and *Clitocybe*, whereas in woodlands, regular fungal fronts may be produced by both saprotrophic and ectomycorrhizal species; a few Ascomycetes and non-sporophore-forming fungi can also generate circular or subtle ring-like patterns [3]. Such circles are typically seasonally observed in organic matter-rich grassland soils under low or moderate disturbance levels [2, 7]. During the vegetative season, the fungal mycelia decompose the soil organic matter as an energy source [8]. The decomposition process of these organic substrates alters the soil pH and releases substantial quantities of micro- and macronutrients into the soil [9]. The dominant fungal species forms a dense, highly branched and interconnected mycelium developing as a fungal front [10, 11], embedded in the soil that becomes paler, relatively more friable, and, in some cases, characterized by a mushroom-like scent and increased hydrophobicity [4, 12, 13]. Recent soil microbiota studies have shown that the effect of fairy ring-forming fungi on vegetation can be accompanied by changes in soil microbial communities [14]. As fungal fronts advance, soil mycobiota diversity may decline markedly, and the fairy ring-forming fungus can become highly dominant, in some case up to 80% of fungal reads relative abundance [15, 16].

Because fairy ring-forming fungi differ in trophic strategy, mycelial-mat development, and decomposition activity, plants responses in presence of fairy ring fungi can vary in time and space, ranging from enhanced growth to vegetation decline [10, 16, 17], although no effect on vegetation are also documented [18]. These vegetation responses have historically formed the basis for a descriptive classification of fairy rings: type I (depressed vegetation over underground active mycelium), type II (a belt of stimulated growth plants over the active mycelium), and type III (no effect on vegetation) [5]. Other variants of type I fairy rings have been also described, including instances with an additional belt of stimulated vegetation on the external side of the fairy ring as well or, on the opposite, a lack of the concentric stimulation after the zone of bare or depressed vegetation [19]. Fairy ring types are vegetation-based, context-dependent categories rather than fixed species-specific traits [3]. They describe symptom expression rather than fungal taxonomy: different fungal species may produce similar type-like responses, while the same species may express different effects depending on soil conditions, vegetation, seasonality, and management. The formation of vegetation patterns in fairy rings is a complex process that may involve phytotoxicity and/or hydrophobicity associated with the colonizing mycelium mat, together with phytostimulation linked to nutrients release from the decomposing aged mycelium [19].

Theoretical model suggests that the centrifugal mycelial expansion requires a self-inhibitory effect acting on the fairy ring fungus after its initial dominance in the soil [19]. In plants, the accumulation of extracellular self-DNA released during litter decomposition has been demonstrated as a possible causal factor of the specific plant–soil negative feedback [20]. Exposure to degraded extracellular self-DNA fragments has also been reported as a general biological phenomenon including fungi [21], although its role in fairy ring dynamics under field conditions remains untested.

**Figure 1:**
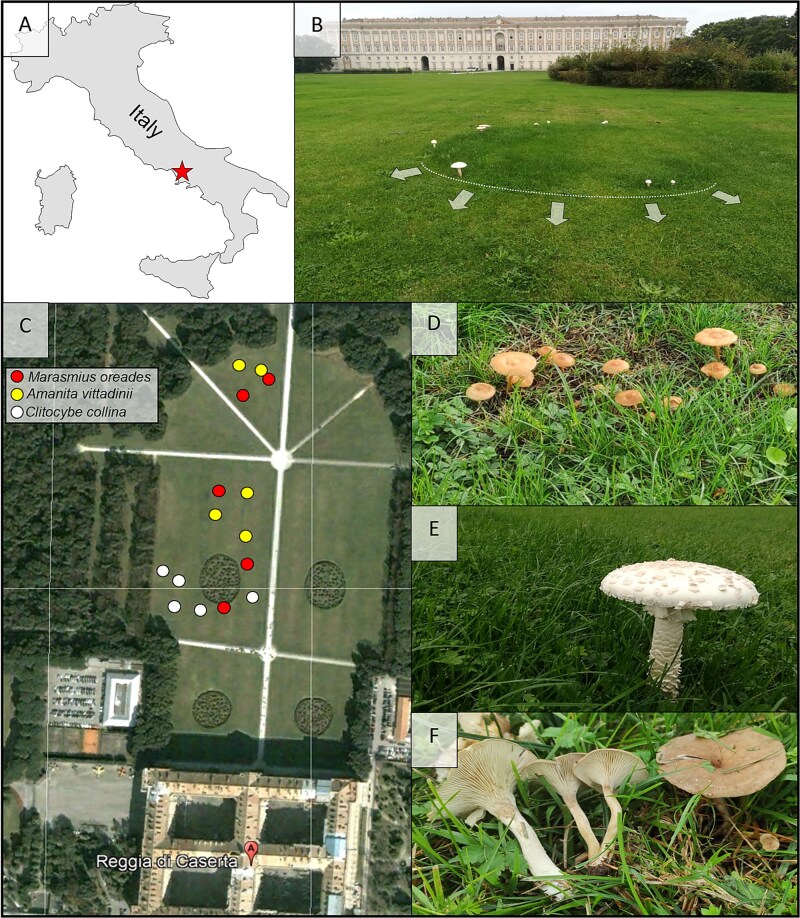
Study site and fairy ring-forming fungi in the managed ornamental grassland of the Royal Palace of Caserta. (A) Location of the study site. (B) Fairy ring in front of the Royal Palace; the line indicates the fungal front, while arrows indicate the widening direction of the ring. (C) Spatial distribution of the sampling plots of fairy rings formed by different fungal species. (D) Sporophores of *M. oreades*. (E) *A. vittadinii*. (F) *C. collina*.

The effects of fairy ring-forming fungi on grassland biota have been mainly investigated in rings showing type I [12, 22] or type II vegetation responses [14, 23]. Although interspecific comparisons of fairy ring-forming fungi have been reported [4, 5], field studies comparing different fairy ring-forming species with the same sampling design and simultaneous plant, soil, and microbial measurements remain scarce. In the present study, each observed type-like pattern was associated with one dominant fairy ring-forming fungal species; therefore, fungal species identity and fairy ring type-like expression could not be statistically disentangled. For this reason, we focused on species-associated responses, while using fairy ring type only as a descriptive framework for the observed vegetation patterns. The simultaneous and contiguous occurrence of fairy rings formed by different fungal species in the managed ornamental grasslands of the Royal Palace of Caserta (Fig. 1) provided an opportunity for a comparative investigation on their functional ecology. This study investigates fairy rings formed by three fungal species, co-occurring within the same managed ornamental grassland. We tested three main hypotheses.

1. We hypothesized that fungal DNA abundance would follow a spatial gradient within each ring, with peak abundance in the active fungal front and persistence in the inner zone after mycelial passage, consistent with the autotoxicity model proposed by Salvatori et al. [19].
2. We hypothesized that each fairy ring-forming species would induce distinct physicochemical soil signatures reflecting species-associated decomposition activity, resulting in divergent soil nutrient and hydro-physical profiles.
3. Building on previous evidence that *Agaricus arvensis* acts as an ecosystem engineer by modulating plant and microbial diversity in species-rich grassland [16], we hypothesized that the fairy ring-forming species investigated here would restructure vegetation and the soil microbiome, with species-associated differences in the magnitude and direction of these effects.

## Material and methods

### Site description, survey design, and soil sampling

The study was conducted in the park of the Royal Palace of Caserta (41°4′24″N, 14°19′35″E, Caserta, Italy), a UNESCO World heritage site (Fig. 1A). The study area is a 12 ha naturalized managed ornamental grassland within the formal estate landscape (Fig. 1B). Management practices include regular mowing and irrigation during the driest months. The local climate is Mediterranean temperate, with hot, dry summers, and mild, wetter winters (Fig. S1). In November 2021, several fairy rings formed by different macrofungal species were observed (Fig. 1C). The fairy ring-forming fungi were identified based on sporophore morphology as *Marasmius oreades, Amanita vittadinii,* and *Clitocybe collina* (syn. *Spodocybe collina*). In this site, these species were associated with type I-, type II-, and type III-like vegetation patterns, respectively. Among more than 30 observed fairy rings, 5 rings per species were selected based on the presence of a regular and clearly identifiable fungal front.

**Figure 2:**
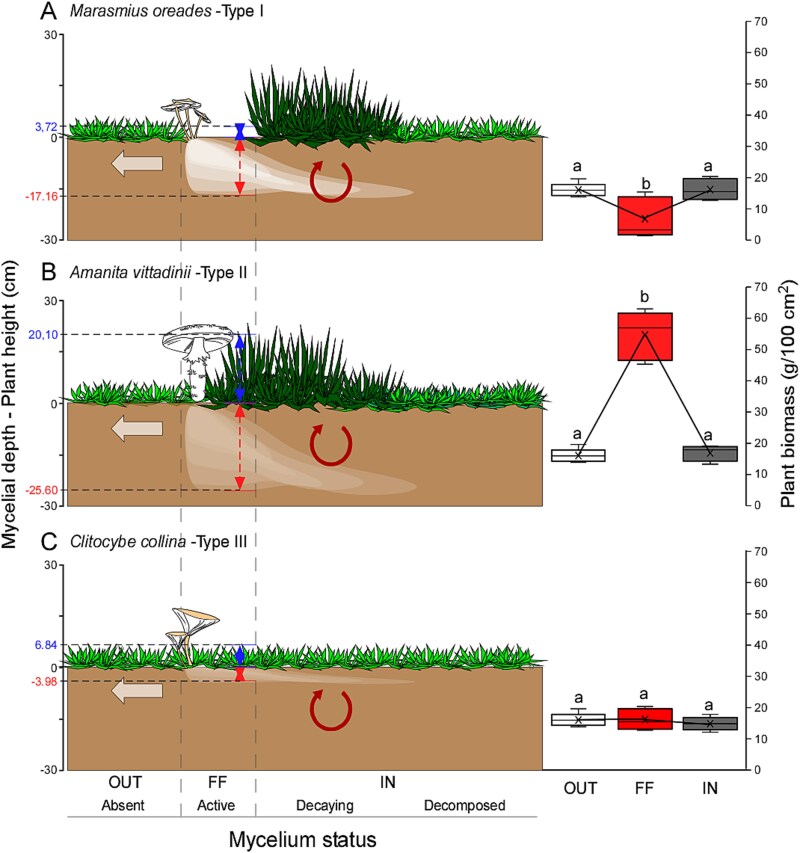
Transverse sections of three fairy rings occurring in the managed ornamental grassland of the Royal Palace of Caserta. (A) Rings formed by *M. oreades*, showing a type I-like vegetation pattern; (B) rings formed by *A. vittadinii*, showing a type II-like vegetation pattern; and (C) rings formed by *C. collina*, showing a type III-like vegetation pattern. OUT: soil and vegetation outside the visible influence of the fairy ring; FF: active fungal front with sporophore emergence; IN: inner post-front zone after mycelial passage. Vertical dashed lines delimit OUT–FF–IN sectors along the transect; the labels below indicate the corresponding inferred mycelial status: Absent, Active, Decaying, and Decomposed. Arrows indicate the direction of fungal front expansion, while circular arrows indicate mycelial decay and associated microbial turnover. For each fairy ring, mycelial depth and vegetation height measured in the FF sector are reported (red dashed arrow = mycelial depth; blue dashed arrow = plant height; values shown in red and blue, respectively). Side boxplots show aboveground plant biomass (g 100 cm^−2^) in OUT, FF, and IN. Lower and upper bounds of the boxplots show the first and third quartiles, the middle line shows the median, and whiskers indicate the interquartile range. Different letters denote significant differences among OUT, FF, and IN zones within each fairy ring-forming species, based on linear mixed-effects models followed by *post-hoc* Student’s *t*-tests (*P* < .05).

To analyze the biotic and abiotic effects of fairy ring-forming fungi, one transect was established across each selected ring, consisting of three 10 cm^2^ plots, labeled as follows: OUT located outside the visible influence of the fairy ring forming fungi; FF corresponding to the active fungal front; IN located inside the ring and representing the post-front zone after mycelial passage (Fig. 2). A total of 35 plots were sampled: 5 OUT plots were collected in the surrounding grassland and 5 replicate FF and 5 replicate IN plots were collected for each fungal species, for a total of 30 ring-associated plots. For each plot, soil was sampled to a depth of ~15 cm using sterilized tools to avoid contamination. Soil samples were air-dried in a laminar-flow microbiological hood, sieved through a 2 mm mesh to remove coarse organic matter and stones, and stored at −80°C until further analysis. Before storing, soils were divided into two aliquots: one for physical and chemical analyses, and one for metagenomic analysis. After the soil sampling, destructive excavation of FF ring zones was performed to assess maximal mycelial depth and distribution.

### Vegetation analysis

The cover of each plant species along the transects was visually estimated. Plant species determination followed the bibliographic repository of Italian flora. Plant cover was recorded using the Braun-Blanquet abundance–dominance scale transformed into percentages cover values as follows: 5 = 85%; 4 = 60%; 3 = 35%; 2 = 15%; 1 = 5%; + = 1%. Plant height was measured in each transect zone.

### Physical and chemical analyses of soils

Soil texture was determined using the pipette method, and water-holding capacity (WHC) was measured according to Alef and Nannipieri [24]. Soil hydrophobicity was assessed using the water drop penetration test (WDPT) [25]. Each test was performed in triplicate. Chemical analyses included measurements of pH (in H₂O) and electrical conductivity in 1:2.5 and 1:5 soil-to-water suspensions, respectively. Organic carbon and total nitrogen content were determined using an CHNS analysis (Micro Elemental Analyzer—UNICUBE®, Elementar, Hesse). Bioavailable elements were extracted using EDTA [26], and their concentrations were measured using inductively coupled plasma-mass spectrometry (ICP-MS, Thermo Scientific iCAP Q ICP-MS).

### Biochemical analyses of soils

The carbon associated to the microbial biomass was determined according to the chloroform fumigation direct extraction method [27]. The soil respiration in terms of CO_2_ released by soil was measured by alkali trap and titration method. The laccase activity was determined by spectrophotometric assay on the oxidation of ABTS [28], whereas the Mn peroxidase activity was tested based on the oxidation of 2,6-dimethoxyphenol method [29]. For each soil sample, the analysis was performed in triplicate for both the activities.

### DNA extraction and metabarcoding

Total microbial DNA was extracted from 200 mg of soil per sample using the DNeasy PowerSoil Kit (Qiagen) following the manufacturer’s protocol. Bacterial communities were assessed via Illumina MiSeq sequencing of the V3–V4 regions of the 16S rRNA gene (~460 bp) using primers S-D-Bact-0341-b-S-17 (5′-CCTACGGGNGGCWGCAG-3′) and S-D-Bact-0785-a-A-21 (5′-GACTACHVGGGTATCTAATCC-3′). Fungal communities were analyzed by sequencing the ITS1–2 spacers of the 18S and 28S rRNA genes (~300 bp), with primers BITS1fw (5′-ACCTGCGGARGGATCA-3′) and B58S3-ITS2rev (5′-GAGATCCRTTGYTRAAAGTT-3′). For bacteria, PCR amplification included 25 cycles of 95°C for 3 min, 95°C for 30 s, 55°C for 30 s, and 72°C for 3 min, with a final extension at 72°C for 5 min. For fungi, PCR included 35 cycles of 95°C for 30 s, 55°C for 30 s, and 72°C for 1 min, with a final extension at 72°C for 5 min. Before Amplicon sequencing, primer performance was preliminarily verified by conventional PCR amplification and agarose gel electrophoresis prior to library preparation, confirming successful target amplification and absence of non-specific bands.

Obtained raw sequences were processed using the DADA2 pipeline in R (v4.5.2), following the standard workflow including quality filtering, trimming, dereplication, error-rate learning, amplicon sequence variant (ASV) inference, paired-end merging, and chimera removal using the consensus method. Taxonomic assignment was performed using the Greengenes database for bacteria and UNITE v.9 for fungi [30, 31]. Alpha diversity metrics were calculated from the original ASV tables. Multivariate analyses of community composition were performed on the ASV tables after Hellinger transformation.

### Data analysis

ASVs assigned to fairy ring-forming fungi were used to estimate the read abundance of the dominant fairy ring-forming fungi along the transect OUT, FF-IN (Zones). For each fairy ring-forming species, variation in read abundance was tested using linear mixed-effects models with Zone as fixed factor and ring identity as random factor. Vegetation, microbial, and soil variables were analyzed using linear mixed-effects models including Zone, Species and, when appropriate, their interaction as fixed effects, with ring identity as a random factor. Analyses were performed in R 4.5.2 using lme4 and lmerTest.

Multivariate patterns were explored by principal component analysis (PCA), including an additional ordination restricted to the FF zone to identify variables associated with each fairy ring-forming species. Fungal and bacterial community compositions were visualized with ternary plots, in which coordinates represented the proportional distribution of ASV read abundance among OUT, FF, and IN zones, and point size was scaled by taxon relative abundance among the 50 most frequent taxa. Differences in fungal and prokaryotic community composition were tested by PERMANOVA on Bray–Curtis dissimilarities using 999 permutations constrained within ring identity.

Hypothesized relationships among fairy ring fungi, soil properties, microbial compartments, and plant biomass were assessed by PLS-SEM. A common conceptual framework was applied across all models, while species-specific SEMs were built by defining the decomposition latent variable according to PCA results. Model structure and indicator allocation are reported in Supplementary Table S1. Further details on model rationale and variables included in the analyses are provided in Supplementary material file.

## Results

### Vegetation responses to fairy ring-forming fungi

Vegetation aboveground biomass patterns across the transect were consistent with type I-, type II-, and type III-like fairy ring responses (Fig. 2). Rings, formed by *M. oreades*, showed a type I-like pattern, with reduced biomass in the FF zone compared with both zone OUT and IN. Rings formed by *A. vittadinii* showed a type II-like pattern with increased biomass in the FF zone, whereas rings formed by *C. collina* showed a type III-like pattern, with no significant biomass differences along the whole transect. Linear mixed-effects models across species (Table S2) showed that plant biomass varied significantly with Species (F = 85.2, *P* < .001) and Zone along the OUT–FF–IN gradient (F = 31.4, *P* < .001). Also, plant richness varied significantly with Species (F = 4.0, *P* = .047) and Zone (F = 8.3, *P* = .002). Plant Shannon diversity showed no significant Species or Zone effects, indicating limited changes in overall plant diversity across species-associated ring patterns and zones.

### Molecular identification and DNA abundance of fairy ring-forming fungi

**Figure 3:**
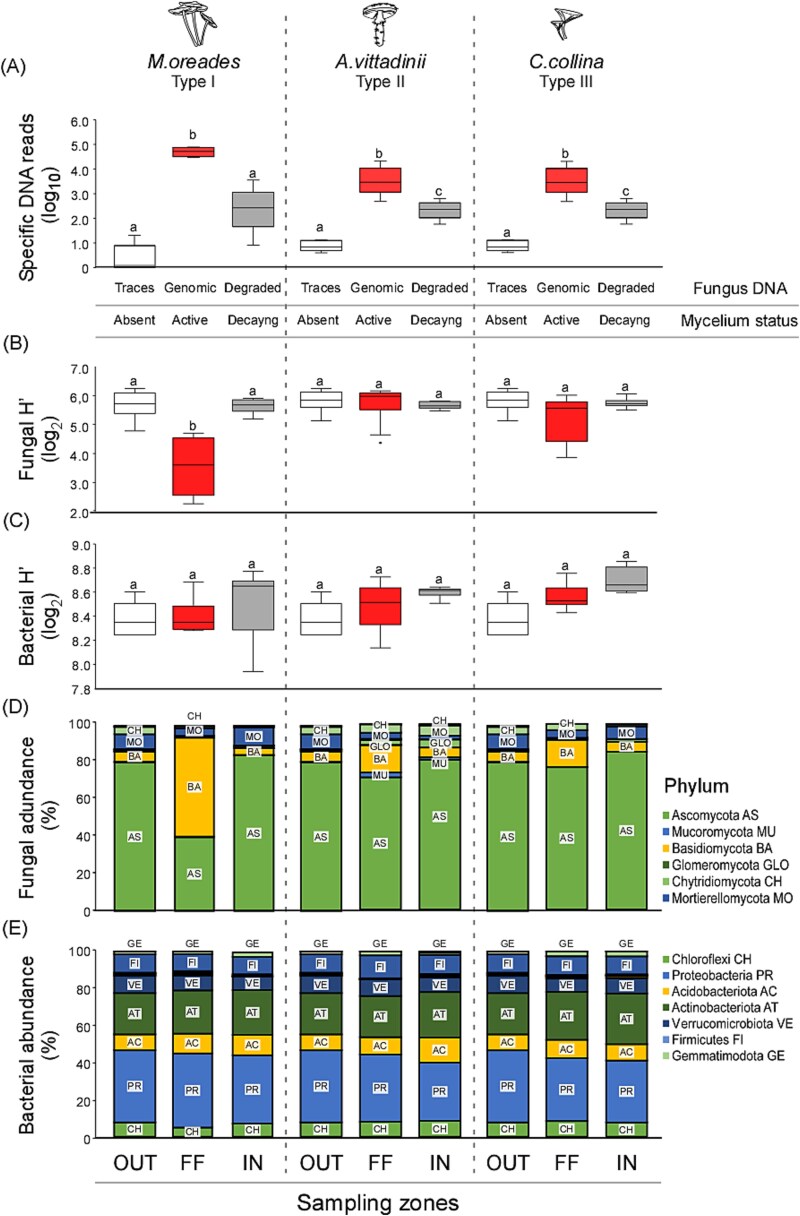
Characterization of fairy ring-forming fungal read abundance and soil microbial diversity across OUT–FF–IN transects. (A) Box-and-whisker plots of reads assigned to *M. oreades, A. vittadinii*, and *C. collina*. Lower and upper bounds of the boxplots show the first and third quartiles, the middle line shows the median, and whiskers indicate the interquartile range. Letters indicate significant differences in read abundance among OUT, FF, and IN zones within each fairy ring-forming species, based on linear mixed-effects models followed by *post-hoc* Student’s *t*-tests (*P* < .05). (B, C) Shannon diversity of fungal and bacterial communities, respectively, across OUT, FF, and IN zones. (D, E) Composition of dominant fungal and bacterial taxa, respectively, across OUT, FF, and IN zones.

ITS-based ASV profiles were used to confirm morphological identification of the three fairy ring-forming fungi and track fairy ring fungal abundance along the OUT-FF-IN transect. Candidate ASVs peaking in the FF zones were inspected, and taxonomic assignments were validated by BLAST searches (Table S3). Reads of the fairy ring-forming fungi showed significant spatial patterns across zones (Fig. 3; Table S2). Read abundance differed among OUT, FF, and IN zones for *M. oreades* (F = 34.9, *P* < .001), *A. vittadini* (F = 2.7, *P* = .043), and *C. collina* (F = 14.7, *P* < .001). For the three species investigated, the highest read numbers were detected in the active mycelial front, whereas negligible reads occurred in the OUT zone and intermediate values were observed in the IN zone. Particularly in the FF zone, *M. oreades* reached the highest reads abundance followed by *A. vittadini* and *C. collina.* Noteworthy, the large amount of DNA recorded from FF zone can be ascribed to genomic content from active mycelia, whereas the DNA recorded from IN zones is mostly constituted by damaged fragments released in the soil by decaying mycelia.

### Change in fungal and prokaryotic communities

Microbial diversity and composition were analyzed across OUT, FF, and IN zones for each fairy ring-forming fungus (Fig. 3; Table S2). For fungi, ASV richness significantly differed among Zones (Table S2). Richness declined in FF for *M. oreades* and *C. collina*, with higher values in IN than FF (Fig. 3B). In *A. vittadinii*, richness differences among zones were smaller and lower richness values extended to IN (Fig. 3B). Shannon diversity differed little among Zones, with the lowest values in FF for *M. oreades* (Fig. 3B; Table S2). For bacteria, diversity metrics (reads, ASV richness, Shannon) were slightly higher in FF and IN than OUT but without statistically significant differences (Fig. 3C; Table S2). Overall, the fungal composition at phylum level was relatively homogeneous across all rings, excepting for the FF of *M. oreades* characterized by a much larger dominance of Basidiomycota (Fig. 3D). No sufficient differences were observed in the bacterial composition for all zones of the transect (Fig. 3E).

**Figure 4:**
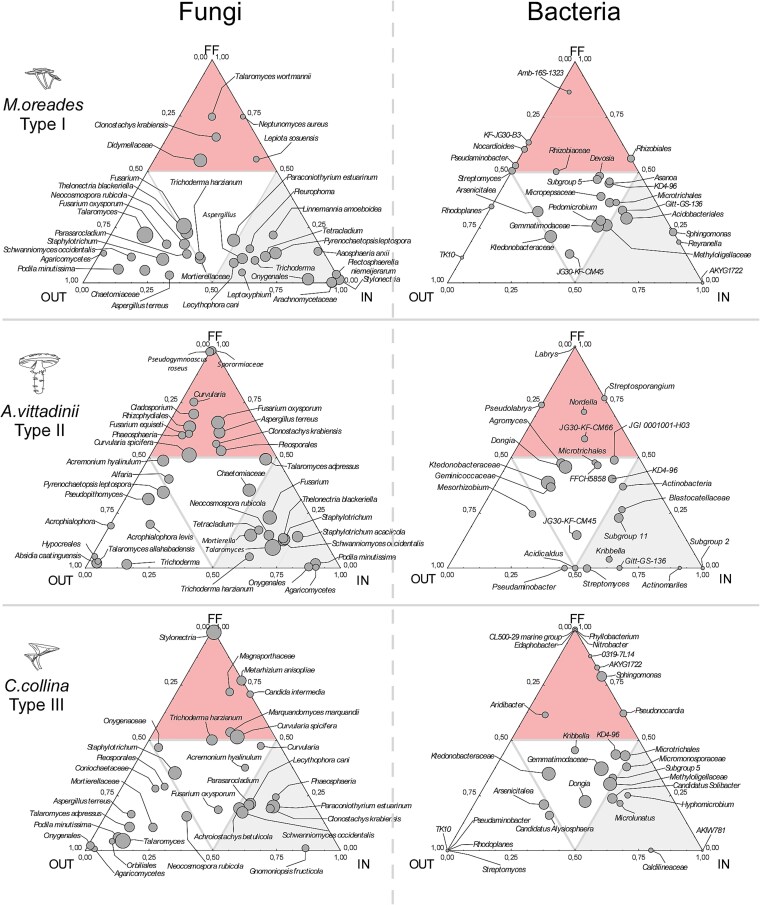
Fungal and bacterial community composition across fairy ring transects in the managed ornamental grassland of the Royal Park of Caserta. Ternary plots show the most frequent fungal taxa (left) and bacterial taxa (right) associated with rings formed by *M. oreades* (top), *A. vittadinii* (middle), and *C. collina* (bottom). Plot coordinates represent the proportional distribution of each taxon among OUT, FF, and IN zones. Circle size is proportional to the average read abundance of each taxon.

At ASV level, fungal communities differed among Zones and according to fairy ring-forming species (Pseudo-F = 6.82; *P* < .001; Tables S4 and S5; Fig. 4). In *M. oreades* rings, the FF zone was characterized by dominance of *M. oreades* mycelium and co-occurrence of taxa including *Clonostachys krabiensis, Talaromyces wortmanii*, and Didymellaceae, whereas OUT and IN contained higher taxonomic richness and included *Trichoderma harzianum* and congeners (Fig. 4). In *A. vittadini* rings, FF contained taxa including *Fusarium oxysporum, C. krabiensis, Aspergillus terreus*, Rhizophydales, *Cladosporium, Curvularia* spp., *Pseudogymnoascus roseus*, and Sporomycetaceae; IN included taxa such as *Podila minutissima, Schwanniomyces occidentalis, Staphylothricum acaciicola*, and *Thelonectria blackeriella* (Fig. 4). In *C. collina* rings, FF contained taxa including *Stylonectria, Metarhizium anisopliae, Candida intermedia*, and Magnaporthaceae; *T. harzianum* occurred in FF as a co-dominant taxon (Fig. 4).

Bacterial communities differed at the ASV level primarily according to fungal species (Pseudo-F = 3.51; *P* < .001) and secondarily according to ring Zone (Pseudo-F = 2.51; *P* < .001; Tables S6 and S7; Fig. 4). In *M. oreades* rings, prokaryotic taxa such as *Amb-165-1323, KF-JG30-B3, Pseudaminobacter*, and *Rhizobiales* increase in percentage contribution in the FF zone compared to OUT. When passing from FF to the IN zone, the community is significantly represented by Acidobacteriales, *Sphingomonas, Reyanella*, and *AKYG1722*. In *A. vittadinii* rings, taxa such as *Labrys, Streptosporangium, Nordella, Pseudolabrys*, and *JG30-KF-CM66* exhibit a slight advantage in the FF zone. Whereas IN differed from FF as the community is characterized by the rise of *Blastocatellaceae, Kribella*, Actinobacteriota, and Acidobacteriaceae of subgroups 11 and 2. In *C. collina* rings, *Aridibacter, Sphingomonas, Pseudonocardia, Edaphobacter*, and *Nitrobacterium* undergo relative heightening in FF compared to the OUT zone. Internally to the fairy ring of *C. collina, Micromonosporaceae, Methyloligellaceae, Hyphomicrobium, Microlunatus*, and subgroup 5 of Acidobacteriaceae produce the buildup of a new community (Fig. 4).

### Chemical and biochemical changes associated with fairy ring fungi

**Figure 5:**
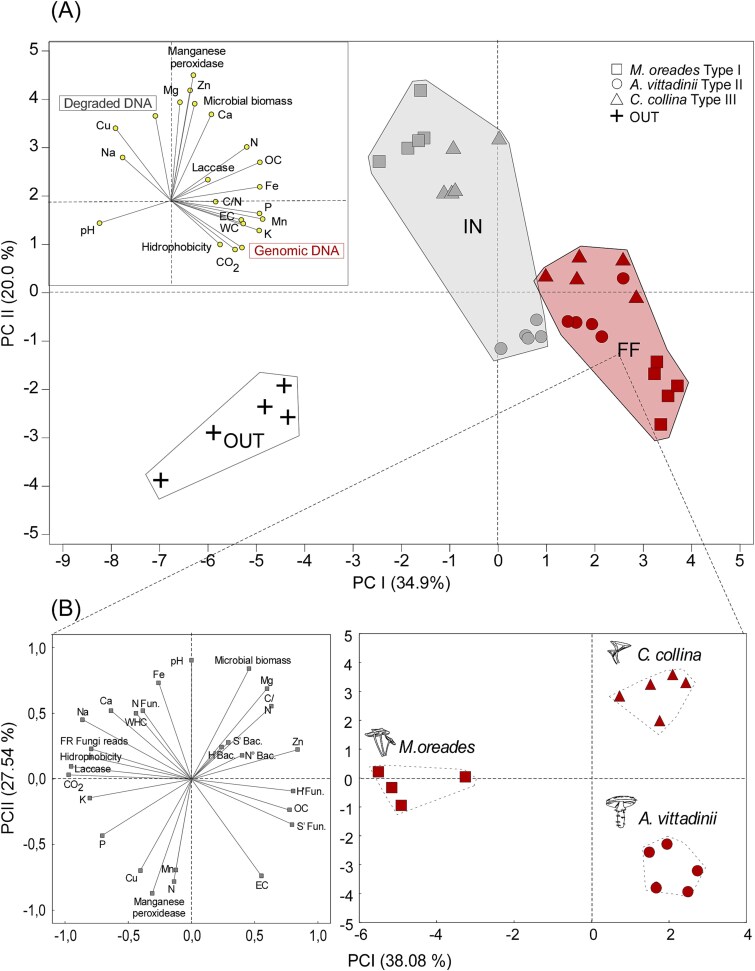
Multivariate characterization of soil chemical, biochemical, and physical parameters across fairy ring transects. (A) Principal component analysis including OUT, FF, and IN zones of the three fairy ring-forming species in the managed ornamental grassland of the Royal Palace of Caserta. The loading plot shows the contribution of variables to the multivariate ordination, while the score plot shows the distribution of sampling points in the space defined by the first two principal components. The legend indicates fungal species and sampling zones. (B) Principal component analysis restricted to FF samples, showing variables associated with the active fungal front of each fairy ring-forming species.

Soil chemical and biochemical profiles varied across ring zones and, for several variables, among fairy ring-forming species (Table S2), with the main multivariate pattern shown in Fig. 5A and detailed values reported in Table 1. Across the three fairy ring-forming species, several variables showed a common zonation pattern, with the largest shifts in the FF zone and intermediate values in the IN zone. This pattern was most evident for CO₂ flux, pH, and electrical conductivity. CO₂ flux increased markedly in the FF, zone with a stronger effects of Zone than Species (Species: F = 144.7, *P* < .001; Zone: F = 347.0, *P* < .001). Soil pH decreased in FF relative to OUT (Zone: F = 137.5, *P* < .001), whereas IN values were generally similar to OUT (Table 1). Electrical conductivity increased in FF and remained higher than OUT in most IN soils (Zone: F = 405.5, *P* < .001). Organic C showed a strong Zone effect (F = 183.3, *P* < .001), reaching up to +40% in FF and +32% in IN relative to OUT. Also, total N varied primarily with Zone (F = 136.6, *P* < .001; Table S2). Several macro- and micronutrients also varied across ring zones, with many showing highest values in FF and species-dependent values in IN.

**Table 1:** Means and standard deviations of soil chemical, physical, and biochemical parameters measured along OUT–FF–IN transects in the managed ornamental grassland of the Royal Park of Caserta. Values are reported for rings formed by *M. oreades, A. vittadinii*, and *C. collina*, associated in this site with type I-, type II-, and type III-like vegetation patterns, respectively. OUT: soil and vegetation outside the visible influence of the fairy ring; FF: active fungal front; IN: inner post-front zone after mycelial passage.

	OUT	FF			IN		
		*Marasmius oreades*	*Amanita vittadinii*	*Clitocybe collina*	*Marasmius oreades*	*Amanita vittadinii*	*Clitocybe collina*
WHC^a^ (%)	58.8 ± 4.89	71.8 ± 3.36	63.4 ± 3.71	70.4 ± 5.69	60.5 ± 5.8	62.2 ± 5.74	60.8 ± 2.34
Microbial biomass (mg/kg)	161.7 ± 16.47	222.8 ± 12.09	241.3 ± 18.28	476.6 ± 21.75	370.1 ± 32.83	187 ± 17.91	357.9 ± 20.12
CO_2_ (mg/kg ds/h)	7.1 ± 1.75	53.4 ± 3.15	17 ± 2.09	19.8 ± 2.92	9.2 ± 2.22	8.6 ± 1.67	9.8 ± 1.09
Hydrophobicity (sec)	1.2 ± 0.59	20.9 ± 13.14	1.4 ± 0.19	7.1 ± 12.54	1.2 ± 0.32	1.4 ± 0.43	1.2 ± 0.32
pH	7.5 ± 0.08	7 ± 0.09	6.9 ± 0.07	7.3 ± 0.1	7.2 ± 0.03	7.4 ± 0.07	7.2 ± 0.09
Manganese peroxidase (mmol)	0.2 ± 0.02	0.6 ± 0.07	0.7 ± 0.06	0.3 ± 0.04	1.3 ± 0.04	0.2 ± 0.02	0.6 ± 0.12
Laccase (mmol)	0.2 ± 0.03	0.6 ± 0.04	0.2 ± 0.04	0.3 ± 0.03	0.2 ± 0.04	0.5 ± 0.04	0.7 ± 0.05
EC^a^ (μS cm^−1^)	70.6 ± 11.7	208.4 ± 18.54	277.4 ± 12.86	211.7 ± 14.12	104.4 ± 11.73	168.5 ± 9.6	98.1 ± 4.83
OC^a^ (g/kg)	38.1 ± 4.03	53.8 ± 3.32	61.1 ± 2.1	59 ± 0.81	50.2 ± 3.59	55.1 ± 3.05	53.4 ± 1.92
N (g/Kg)	4.4 ± 0.11	6 ± 0.26	6.3 ± 0.47	5.6 ± 0.43	5.8 ± 0.4	5.6 ± 0.24	5.8 ± 0.48
C/N^a^	8.7 ± 0.83	9 ± 0.25	9.8 ± 0.64	10.7 ± 0.83	8.7 ± 0.82	9.8 ± 0.4	9.2 ± 0.55
K (mg/kg)	1332 ± 135.94	1776.7 ± 110.92	1538.2 ± 67.75	1535.6 ± 105.73	1481.2 ± 99.29	1760.5 ± 91.3	1451.7 ± 104.25
Ca (mg/kg)	5244.1 ± 437.15	6203.8 ± 232.91	5745.2 ± 197.3	6200.6 ± 265.92	6632.5 ± 173.34	6291.2 ± 372.77	6253 ± 253.74
Na (mg/kg)	129.2 ± 10.43	122.4 ± 1.1	87 ± 1.94	103.8 ± 4.57	161 ± 4.84	85.4 ± 2.21	107.5 ± 2.99
Mg (mg/kg)	462.3 ± 52.27	485.3 ± 43.16	507.7 ± 24.05	600.8 ± 24.91	577.8 ± 1.69	535.6 ± 9.47	608.5 ± 1.78
P (mg/kg)	8.1 ± 1.78	16.5 ± 1.21	14.1 ± 0.88	12.9 ± 1.08	11.1 ± 0.88	16.5 ± 0.95	12.3 ± 1.05
Fe (mg/kg)	163.6 ± 23.44	270 ± 19.19	237.2 ± 13.04	278 ± 20.55	226.6 ± 3.61	220.7 ± 0.78	201.3 ± 2.95
Mn (mg/kg)	91.5 ± 7.95	170.2 ± 10.2	184 ± 15.91	154.3 ± 20.72	100.1 ± 12.96	142.7 ± 1.78	138.1 ± 0.17
Zn (mg/kg)	8.2 ± 2.02	9.5 ± 0.26	11.5 ± 0.38	12.1 ± 0.69	13.5 ± 0.15	11 ± 0.16	13.5 ± 0.81
Cu (mg/kg)	12.9 ± 0.97	11.3 ± 1	11.6 ± 1.1	9.7 ± 1.18	14.9 ± 0.41	10.2 ± 0.08	13.8 ± 0.77

^a^Water-holding capacity (WHC), electrical conductivity, organic carbon, and carbon-to-nitrogen ratio.

*P* showed significant effects of both Zone and Species, with a stronger effect of Zone (Zone: F = 108.0; *P* < .001; Species: F = 5.6; *P* = .019), whereas K varied significantly only with Zone (F = 62.0; *P* < .001). Fe also varied primarily with Zone (F = 97.6, *P* < .001; Table S2). A subset of variables showed stronger species-associated differences than the variables above. Hydrophobicity showed a significant Species × Zone interaction (F = 7.3, *P* < .001), with the largest increase observed in the FF zone of *M. oreades*, where values were approximately 20-fold higher than in OUT soils (Table 1). Extracellular enzyme activities differed across zones and among fairy ring-forming fungi. Laccase showed a significant Species × Zone interaction (F = 209.3, *P* < .001), with the highest values in FF of *M. oreades*. Mn peroxidase also showed strong Species, Zone effects (Species: F = 127.7, *P* < .001; Zone: F = 419.7, *P* < .001), with the strongest increases in *A. vittadinii* and with elevated values in IN zone of *M. oreades* (Table 1, Table S2). Genomic and degraded DNA showed a consistent zone-dependent pattern across the three species, with genomic DNA peaking in the FF zone and degraded DNA peaking in the IN zone.

The PCA restricted to FF samples (Fig. 5B) showed a clear separation among the active mycelial fronts of the three fairy ring-forming species. *M. oreades* was mainly associated with fairy ring fungal reads, hydrophobicity, laccase activity, and CO₂ flux, whereas *A. vittadinii* and *C. collina* were separated along PC2: *A. vittadinii* was associated with higher EC and Mn peroxidase activity, while *C. collina* was associated with higher pH, microbial biomass, Mg, and C/N.

### PLS-SEM integration of plant–microbiome–soil responses

**Figure 6:**
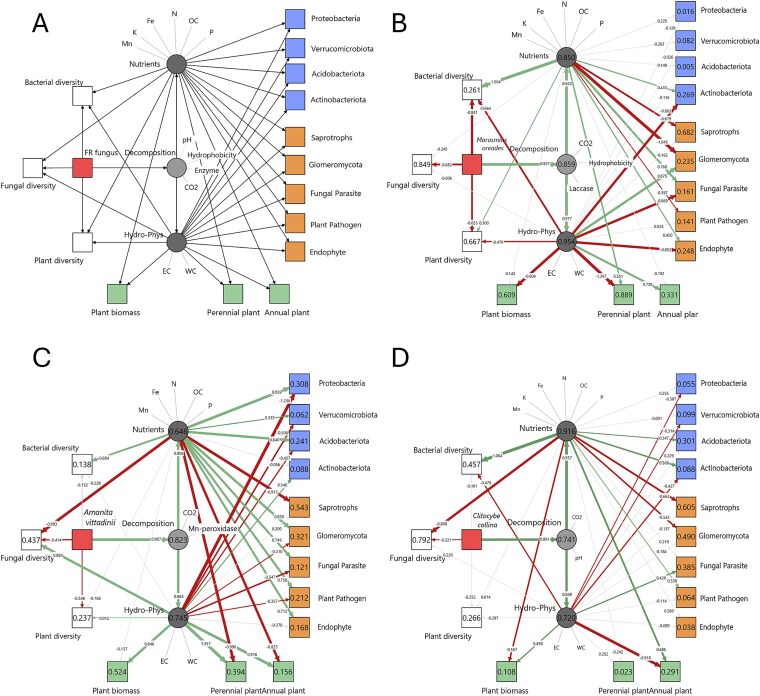
Partial least squares structural equation models depicting causal relationships among fairy ring-forming fungi, soil properties, biotic compartments, and plant biomass in the managed ornamental grassland of the Royal Park of Caserta. Panel (A) shows the global baseline model including the variables tested. Panel (B) represents the species-specific model for rings formed by *M. oreades*, associated in this site with a type I-like vegetation pattern. Panel (C) represents the species-specific model for rings formed by *A. vittadinii*, associated with a type II-like vegetation pattern. Panel (D) represents the species-specific model for rings formed by *C. collina*, associated with a type III-like vegetation pattern. Latent variables are represented by circles and observed variables by rectangles. Directed arrows indicate hypothesized relationships among constructs. Standardized path coefficients are reported on the arrows. Arrow color indicates the direction of the relationship, while arrow thickness is proportional to the magnitude of the path coefficient. Paths with coefficients between −0.3 and 0.3 are shown in gray and were considered weak effects. Values reported within latent and observed variables correspond to adjusted R^2^ values. Observed variables are color-coded according to ecological compartment: fairy ring fungal read abundance, plant-related variables, fungal guilds, bacterial phyla, and diversity metrics.

Species-specific PLS-SEM models were fitted to link fairy ring fungal read abundance to Decomposition and to two latent soil-property constructs, Hydro-physical properties and Nutrients, and subsequently to microbial compartments and plant biomass (Fig. 6; Tables S1, S8, and  S9). Fairy ring fungal abundance was associated with Decomposition in the three species-specific model. The Decomposition construct was represented by different indicator sets in the three species-specific model: CO₂ flux, laccase, hydrophobicity for *M. oreades*; CO₂ flux, Mn peroxidase for *A. vittadinii*; and CO₂ flux and pH for *C. collina*. In the *M. oreades* model (corresponding to type 1-like effect), plant diversity declined with fungal abundance (β = −0.653; R^2^ = 0.67). Total and perennial biomass were negatively associated with Hydro-physical properties (β = −0.904, R^2^ = 0.61; β = −1.397, R^2^ = 0.89, respectively), whereas annual biomass was positively associated with the same construct (β = 0.738; R^2^ = 0.33). In the *A. vittadinii* model (type II-like effect on vegetation), total biomass was positively associated with Hydro-physical properties (β = 0.846; R^2^ = 0.52), whereas plant diversity metrics showed low explained variance. In the *C. collina* (type III-like), plant-related paths were negligible. Saprotrophic fungi were negatively associated with Nutrients in the three species-specific models (type I: β = −0.679, R^2^ = 0.68; type II: β = −0.912, R^2^ = 0.543; type III: β = −0.662, R^2^ = 0.60). Glomeromycota were associated with Nutrients in *A. vittadinii* (β = 0.830; R^2^ = 0.32) and *C. collina* rings (β = −0.543; R^2^ = 0.49), whereas fungal parasites were associated with Hydro-physical properties only in *C. collina* rings (β = 0.429; R^2^ = 0.385). Among bacterial phyla, Actinobacteriota were associated with Hydro-physical properties in *M. oreades* model (β = −0.863; R^2^ = 0.26), Proteobacteria in *A. vittadinii* rings were associated with both Hydro-physical properties and Nutrients (β = −1.236 and β = 0.959; R^2^ = 0.308), and Acidobacteriota in *C. collina* rings were associated with Nutrients (β = 0.347; R^2^ = 0.301). Bacterial diversity was positively associated with Nutrients independently by species-specific effect (*M. oreades*: β = 1.504; *A. vittadini*: β = 0.684; *C. collina*: β = 1.063), although explained variance was substantial only in *C. collina* model (R^2^ = 0.457).

## Discussion

### Fairy ring development and formation

We hypothesized that fungal DNA abundance would follow a spatial gradient within each fairy ring, with peak accumulation at the active mycelial front and persistence of degraded DNA fragments in the inner zone after mycelial passage, consistent with the autotoxicity framework proposed by Salvatori et al. [19]. The observed distribution of reads supports this expectation across all three fairy ring-forming species analyzed, with consistently higher abundance in the fungal front and detectable persistence in inner zones relative to outer unaffected areas.

Similar spatial dynamics have previously been reported for *A. arvensis* and *Calocybe gambosa* [15, 16], where the dominant fairy ring fungi accounted for 70%–80% of total fungal reads in the active front, followed by a marked decline in inner sectors but with a residual signal of the fungus forming the front. In those systems, the reduction in dominant fungal abundance in post-front zones coincided with the emergence of microbial taxa exhibiting chitinolytic activity, consistent with the decomposition of senescent mycelial residues and turnover of fungal biomass.

The formation of circular fungal fronts has long raised the question of why colonies expand as rings rather than disk-like structures [4]. Early explanations emphasized nutrient depletion within inner zones; however, in a soil inversion experiment, *Clitocybe nebularis* was unable to regrow in soil previously colonized by its own mycelium despite restored organic matter levels [11], suggesting that resource depletion alone cannot fully account for ring formation. Within this broader context, self-inhibition mediated by extracellular self-DNA has been proposed as a general ecological mechanism underlying spatial pattern formation [20]. Self-DNA has been experimentally shown to exert species-specific inhibitory effects across a wide range of biological systems, including plants, animal, and yeasts, where accumulation of conspecific DNA can impair growth and proliferation [21]. As a water-soluble compound released during cellular turnover, extracellular DNA represents a plausible autotoxic factor capable of generating localized negative feedback.

**Figure 7:**
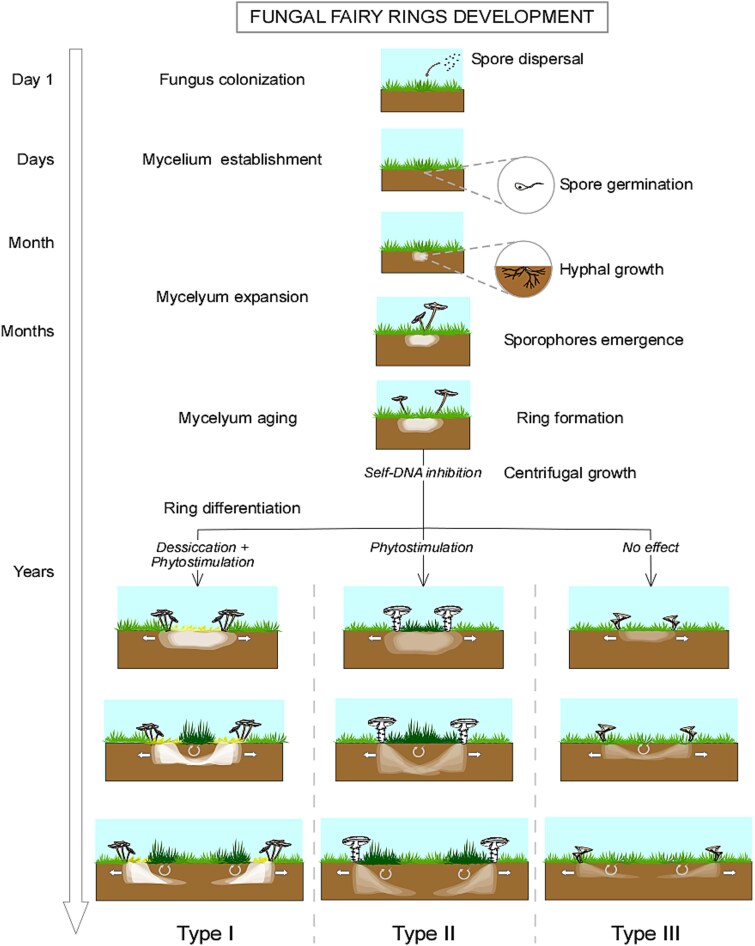
Site-specific conceptual framework summarizing the developmental patterns and associated processes observed in the three fairy rings investigated in the managed ornamental grassland of the Royal Park of Caserta. After fungal establishment, the circular distribution of sporophores and the spatial pattern of fungal reads indicate centrifugal mycelial expansion from the active fungal front toward external soil. Residual fungal reads in the inner post-front zone reflect senescent mycelial residues and support the role of degraded extracellular self-DNA in self-inhibitory dynamics. Rings formed by *M. oreades* showed a type I-like pattern, with reduced vegetation associated with the active fungal front. Rings formed by *A. vittadinii* showed a type II-like pattern, with vegetation stimulation associated with the fungal front. Rings formed by *C. collina* showed a type III-like pattern, with negligible vegetation response at the scale of observation. Mycelial decomposition and nutrient turnover are indicated by circular arrows.

Although our field data do not directly quantify extracellular self-DNA concentrations, the recurrent spatial persistence of fungal DNA in inner zones across multiple species and studies is consistent with both predicted and observed accumulation of senescent mycelial residues following front passage. Together, these findings provide comparative field-based empirical support for a spatially structured self-inhibitory mechanism contributing to ring formation dynamics and support the mechanistic plausibility of the modeling framework proposed by Salvatori et al. [19] under natural field conditions. The conceptual framework illustrated in Fig. 7 integrates the shared developmental dynamics observed in the fairy rings of our study site, highlighting a common centrifugal growth pattern associated with the spatial distribution of degraded fragments of fungal DNA.

### Decomposition dynamics and enzymatic specialization within the fairy ring syndrome

In line with our second hypothesis, we predicted that each fairy ring-forming species would induce distinct physicochemical soil signatures reflecting species-specific decomposition pathways, resulting in divergent nutrient and hydro-physical profiles among fairy ring types. Our results support this prediction: while all rings shared a common decomposition-driven shift at the advancing front, the magnitude and structure of enzymatic, nutrient, and hydro-physical responses differed among *M. oreades, A. vittadinii*, and *C. collina*.

As the fungal front advances, organic matter decomposition intensifies, producing a marked increase in CO₂ flux that indicates a metabolic burst associated with active mycelial growth and substrate oxidation. Concomitantly, soil pH declines, an established feature of fairy rings [8, 17, 18, 32], likely driven by proton release and organic acid production during mineralization. Together, CO₂ increase and acidification define the biochemical core of the fairy ring syndrome.

Dense mycelial mats at the front release oxidative enzymes, particularly laccases and Mn peroxidases, which drive lignocellulosic transformation. Laccases oxidize a wide range of phenolic and aromatic substrates and have been repeatedly implicated in lignin degradation within fairy rings [9, 13]. In our system, laccase activity was strongly associated with *M. oreades*, consistent with observations from German grasslands [9].

Mn peroxidases, by contrast, are more substrate-specific among basidiomycete ligninolytic enzymes [33]. Here, Mn peroxidase activity was strongly associated with *A. vittadinii* and tended to increase within the rings of *M. oreades* and *C. collina*. Notably, Mn peroxidase-related activity was often detected in inner zones where basidiomycete dominance had already declined, consistent with the idea that laccase-driven oxidation may prevail in early phases, whereas Mn peroxidase-dependent pathways become more evident later or persist as residual signals [9]. Although comparative data on enzyme persistence in soils are limited and stability depends on pH and moisture [34], a parsimonious interpretation is that both enzymes contribute to decomposition with different temporal intensities, with Mn peroxidase-dependent processes becoming detectable as substrates shift toward more recalcitrant fractions.

In parallel with enzymatic activity, mineralization mobilizes nutrients and metal cofactors. Increased Fe and Mn are consistent with their central role in oxidative metabolism [35, 36], and Fe enrichment may be supported by siderophore-mediated sequestration in the extramycelial matrix [37], sustaining high metabolic demand within vegetative mycelium [15]. P and K enrichment further indicate intensified nutrient mobilization, as widely reported in fairy ring systems [12, 14, 17, 38]. Although ammonia and nitrite were not measured, the increase in total nitrogen and the enrichment of copiotrophic taxa are compatible with localized nitrogen transformations accompanying front expansion [3, 12, 15, 39]. The establishment of this hypertrophic zone results in strong competitive dominance by the fairy ring-forming fungi [40]. Accordingly, fungal diversity declines sharply at the active front, and in our study, the dominant mycelium represented 20%–55% of the total fungal community. Hydro-physical responses further contributed to divergence among fairy ring species, consistent with differential hydrophobin production and soil-matrix modification [5, 41]. Beyond their defensive role, hydrophobins can facilitate colonization of hydrophobic substrates and protect sporophore development [42].

Overall, these results indicate that all three fungi share a common decomposition-driven transformation of soil chemistry, but differ in the balance between laccase- and manganese-dependent pathways, nutrient mobilization patterns, and hydro-physical effects.

### Ecosystem engineering and divergent biotic outcomes across fairy ring fungi

Building on previous evidence that *A. arvensis* can act as an ecosystem engineer by modulating plant and microbial diversity in species-rich grassland [16], we hypothesized that the fairy ring-forming species investigated here would restructure vegetation and soil microbial communities, with species-associated differences in the magnitude and direction of these effects. The results support this hypothesis in a context-dependent way. All three fairy ring-forming species showed a clear decomposition signal and a consistent capacity to restructure the soil mycobiome, but the translation of these soil changes into plant and bacterial responses differed among species. Thus, the observed type I-, type II-, and type III-like vegetation patterns should be interpreted as species-associated responses in this managed grassland rather than as general ring-type effects.

All fairy ring fungi showed a strong decomposition signal and a consistent capacity to restructure the soil mycobiome, whereas the magnitude and direction of plant and bacterial responses differed among fungal species and decomposition strength. Fairy ring-forming fungi can be interpreted as ecosystem engineers because they modify soil physicochemical conditions in spatially structured patterns that alter habitat suitability and resource availability for other organisms [10, 16]. PLS-SEM supports this mechanistic view by linking fairy ring-forming fungi abundance to decomposition and by resolving two mediation pathways, Nutrients and Hydro-physical properties, through which soil modification propagates to microbial compartments and plant responses. The engineering imprint was strongest on fungi, more moderate on bacteria, and highly variable on vegetation.

*M. oreades* rings showed the clearest vegetation response conducible to a type I-like effect, with reduced plant diversity and pronounced negative effect on plant biomass. These responses are consistent with a prominent role of hydro-physical alteration, including hydrophobin-mediated hydrophobization, which can reduce infiltration and reshape water distribution, thereby generating localized stress at the rooting interface even when bulk soil water content increases compared to unaffected zones [43]. In *A. vittadini*, rings showed a stimulation pattern rather than turnover in plant community. Vegetation changes were mainly expressed as increased biomass of dominant grassland components without clear diversity shifts, consistent with a nutrient-driven productivity pulse as repeatedly reported in several studies [39]. In our case, the causal model explained plant stimulation through the increase in EC and water content compared to nutrient that is more in line with the observation made on *A. arvensis* in English grassland where, despite stimulation, nutrient adsorption impairment was observed [17]. *C. collina* rings showed null vegetation responses at the scale captured by our survey. This indicates that the strength of decomposition signals does not translate into a measurable plant effects in fairy ring pattern.

Across all fungal species, the most consistent biotic outcome was the restructuring of fungal communities. The fungal fronts generated a simplified zone dominated by the fairy ring-forming fungi mycelium, with marked declines in fungal diversity and compositional shifts typical of dominance-driven assembly [40, 44, 45]. Beyond this shared dominance effect, each species assembled distinct fungal consortia in FF and IN zones, consistent with a transition from active-front dominance to legacy-driven recolonization.

In line with previous observations in *A. arvensis* and *C. gambosa* rings [15, 16], taxa compatible with mycoparasitism or chitinolysis occurred around dense mycelial mats. In our system, antagonists included *C. krabiensis* in *M. oreades, Cladosporium* in *A. vittadini*, and chitinolytic or entomopathogenic taxa including *Metarhizium* in *C. collina* [46–50]. Potential plant pathogenic opportunists were also detected in species-specific ways, for instance *Fusarium, Stylonectria*, and *Curvularia*, consistent with transient niche opening around the fungal front [51–53], as previously proposed for *C. gambosa* rings [15].

A key SEM outcome concerned the fungal functional compartment and highlighted patterns not uniformly shared across species. Glomeromycota showed contrasting, species-dependent responses along the nutrient-related pathway, with a positive association in *A. vittadinii* rings. This can be due to the increase in plant growth in verdant vegetation belt as also observed in *A. arvensis* [16]*. C. collina* rings displayed a distinctive signal for fungal parasitism. In *C. collina*, this functional signal is coherent with the occurrence of *Marquandomyces marquandii* that is reported as a specific pathogen of Hygrophoraceae and representing consistent link with the recent taxonomic repositioning of *C. collina* within Hygrophoraceae [54].

Bacterial communities showed little or no changes. Diversity metrics changed only slightly, and compositional changes were weaker than in fungi, consistent with bacteria responding indirectly to mineralization products and mycelial residues. Hyphomicrobiales taxa recurrently appeared in association with mycelial mats across fairy ring-forming species, consistent with copiotrophic strategies and with roles in nitrogen transformations, including diazotrophy and nitrification [55, 56]. Actinomycete lineages with recognized chitinolytic or antimycotic potential were also observed, consistent with their hypothesized role as microbial recyclers on senescent fungal mats [57, 58].

Together, these patterns indicate that fairy ring fungi consistently engineer soil conditions through decomposition, but the translation into plant and bacterial responses depends on the fungal species and on the decomposition pathway. A key caveat is that fairy ring typology should not be interpreted as a fixed species trait. *M. oreades* is known to generate both types I and II outcomes, and historical evidence suggests that the expression of depressed versus stimulated rings depends on climatic context, particularly precipitation regimes [3]. Therefore, the contrasting patterns observed here should be viewed as context-dependent expressions of ecosystem engineering rather than immutable species signatures. Under drier years, hydro-physical alteration, including hydrophobization, may become sufficiently strong to shift plant responses toward a type I-like [19]. Under wetter years, higher water inputs could weaken hydrophobic effects and shift outcomes toward type II-like stimulation. This implies that the strongest engineering footprint on vegetation may be intermittent, emerging when precipitation and or effective hydrophobin production and persistence cross threshold conditions.

## Conclusions

The analysis of dominant fungal DNA sequences recorded along the OUT–FF–IN transects revealed a recurrent mycelial spatial pattern in the three fairy ring-forming fungi investigated at the Royal Palace of Caserta study site. DNA assigned to the fairy ring-forming fungal species peaked in the active fungal front and remained detectable in inner soils, reflecting the persistence of fungal signal in the soil after the mycelial passage. This pattern is compatible with the reported self-inhibitory effect of extracellular degraded DNA, thus providing a logical explanation for the origin of the circular fairy ring pattern, driven by the centrifugal growth of active mycelia away from the senescent or decaying zone of the same fungus. In our study site, the transient dominance of the active mycelium at the fungal front, followed by residual fungal reads in the inner zone, supports a site-specific conceptual interpretation of centrifugal ring development (Fig. 7).

In the studied managed grassland, all fairy ring-forming fungi expressed a common functional syndrome at the advancing front, consistently increasing CO₂ flux, soil acidification, and nutrient mobilization. However, a more detailed examination of front dynamics also revealed some clear species-associated effects, indicating a role of different decomposition strategies of the dominant fungi differently affecting the surrounding biota.

In conclusion, only the case of ring formed by *M. oreades*, forming type I-like fairy rings, can be recognized as an ecosystem engineer, because of its major effects on both vegetation and soil microbial community. Differently, the other fungal species, despite the presence of milder effect on the fungal community, did not produce major impacts on the grassland biota.

## Supplementary Material

Supplementary_material_revised_R3_ycag177

## Data Availability

Data supporting the findings are available in SRA with accession number PRJNA1464561 and in Table 1.
