# Supplementary material for: The fellowship of the ring: species-associated effects of fairy ring fungi on soil, microbiota, and vegetation in a managed ornamental grassland
Source: ISME Commun. 2026 Jun 22;6(1):ycag177. doi: 10.1093/ismeco/ycag177 (PMC13356810; doi:10.1093/ismeco/ycag177)
Supplement: Supplementary_material_revised_R3_ycag177 [file supplementary_material_revised_r3_ycag177.docx]

# **Title:**

The fellowship of the ring: Species-associated effects of fairy ring fungi on soil, microbiota and vegetation in a managed ornamental grassland

**Running Title:**

Fairy rings in managed grassland

## **Authors:** Maurizio Zotti^1^*, Mohamed Idbella^2^, Giuseppina Iacomino^1^, Astolfo Zoina^1^, Roberta Di Lecce^1^, Alberto Minelli^4^, Maria Antonietta Rao ^1^, Giuliano Bonanomi^1,3^, Stefano Mazzoleni^1,3^*

**Affiliations:**

^1^Department of Agricultural Sciences, University of Naples Federico II.

^2^College of Agriculture and Environmental Sciences, AgroBioSciences (AgBS) program, Mohammed VI Polytechnic University, Ben Guerir 43150, Morocco.

^3^Task force of Microbiome Study.

^4^ Department of agricultural and food sciences, Alma Mater Studiorum, University of Bologna.

***Correspondence:**
Maurizio Zotti

Department of Agricultural Sciences

University of Naples Federico II

Piazza Carlo di Borbone, 1

80055 Portici, Naples, Italy

Email: maurizio.zotti@unina.it

Stefano Mazzoleni

Department of Agricultural Sciences

University of Naples Federico II

Piazza Carlo di Borbone, 1

80055 Portici, Naples, Italy

Email: stefano.mazzoleni@unina.it

Figure S1: Cumulative precipitation (mm) per month in the Vitulazio (CE) meteo station (4 km from sampling site) in years 2020. 2021 and 2022. Solid black line indicates the year of appearance of fairy rings.

*Data analysis*

Amplicon sequence variants (ASVs) corresponding to fairy ring-forming fungi were selected to quantify fungal DNA abundance across ring zones. For each fairy ring-forming species, variation in read abundance along the transect (OUT, FF, IN) was analyzed using linear mixed-effects models including Zone as fixed factor and ring identity as a random intercept. For all other vegetation, microbial, and soil variables, linear mixed-effects models were fitted including Species and Zone as fixed factors and ring identity as a random intercept to account for the nested sampling design (multiple zones sampled within rings). Statistical inference for fixed effects was based on type 3 ANOVA with Satterthwaite’s approximation for denominator degrees of freedom. To quantify the magnitude of hierarchical dependence, the intraclass correlation coefficient (ICC) was calculated for each model as the proportion of total variance attributable to between-ring variability. All statistical analyses were performed in R version 4.5.2 (R Core Team) using the packages *lme4* and *lmerTest*.

Ordination of variables obtained from the study were visualized in Principal Component Analysis (PCA). Subsequently, PCA ordinations were restricted to FF zone only to assess the variables associated to each fairy ring forming fungus. Ternary plots were used to visualize fungal and bacterial community composition, with the three axes corresponding to the zones along the transect (OUT, FF, and IN). For each taxon, coordinates were defined by the percentage distribution of its abundance among the three zones, calculated from raw ASV read counts as the proportion of reads in each zone relative to the total reads of that ASV. Dot size was scaled according to the relative abundance of each taxon within the community, calculated across the 50 most frequent taxa represented in the plot. Multivariate differences in fungal and prokaryotic community composition were evaluated using permutational multivariate analysis of variance (PERMANOVA) based on Bray–Curtis dissimilarity matrices. Analyses were conducted with 999 permutations. To account for the hierarchical sampling design, permutations were constrained within ring identity, thereby avoiding pseudoreplication due to repeated sampling of OUT, FF and IN zones within each fairy ring.

To further inspect causal relationships among fairy ring forming fungi, soil properties, microbial compartments, and plant biomass, PLS-SEM (Partial Least Squares Structural Equation Modelling) were carried out. Specifically, For bacterial communities, microbial compartments were described at the phylum level, as this taxonomic resolution captures broad functional ecological strategies within Prokaryotes. For fungi, functional information was obtained by applying FUNGuild classification to metagenomic data [32]. Plant responses to fairy rings fungi activity were broadly quantified using total aboveground biomass, and additionally partitioned biomass of dominant perennial species and opportunistic annual species to quantify species turnover within plant community as suggested in Zotti et al. in 2020 [16].

PLS-SEM analyses were conducted using a common conceptual framework shared across all models, whereas model estimation was performed on species-specific SEMs designed to capture distinct fungal decomposition strategies. In detail, fairy ring forming fungus-specific SEMs were built by customizing the latent variable decomposition. The selection of observed indicators defining decomposition in each model was guided by the results of the Principal Component Analysis (Fig. 5B), which identified the variables most strongly associated with each fairy ring–forming species.

For *M. oreades* fairy rings, the latent variable decomposition was defined by the combined contribution of soil CO₂ flux, laccase activity, and soil hydrophobicity. For *A. vittadinii* I fairy rings, decomposition was defined by soil CO₂ flux and manganese peroxidase activity. For *C. collina* fairy rings, the decomposition latent variable was defined by soil pH and CO₂ flux. The variables included in each model and their allocation to latent constructs are reported in Supplementary table S1.

PLS-SEM estimation was conducted using the path weighting scheme, in order to explain the variance in endogenous constructs. Initial outer weights were set to 1.0. The maximum number of iterations was set to 3000, with a convergence stop criterion of 10⁻⁷. All models converged within the predefined iteration limit, and all calculations were successfully completed.

The reliability and convergent validity of latent constructs were assessed using composite reliability (ρₐ) and Average Variance Extracted (AVE) (Supplementary table S8). Adjusted R² values were calculated for each endogenous latent and observed variable to quantify the proportion of variance explained by the model while accounting for differences in model complexity. Global model fit was evaluated using SRMR and d_ULS values derived from the saturated models (Supplementary table S9). Prior to analysis, all data were log-transformed to correct for heteroscedasticity. Analyses were performed using Statistica 10, Primer-7 and Smart-PLS software.

Table S1**.** Observed variables included in the species-specific Partial Least Squares Structural Equation Models (PLS-SEM) developed for Type I (*M. oreades*). Type II (*A. vittadinii*). and Type III (*C. collina*) fairy rings. The table reports all measured variables used in each model. grouped by ecological and functional category. The presence of a variable in a given model is indicated by X. whereas a dash (–) indicates that the variable was not included. Variables marked with an asterisk (*) correspond to the observed indicators used to define the latent construct decomposition. which was custom-parameterized for each fairy ring type based on species-specific functional strategies identified by Principal Component Analysis (PCA; Fig. 5).

| **Variable category** | **Observed variable** | ***M. oreades*** | ***A. vittadinii*** | ***C. collina*** |
| --- | --- | --- | --- | --- |
| Fungal driver | *M. oreades* abundance | X | - | - |
|  | *A. vittadinii* abundance | - | X | - |
|  | *C. collina* abundance | - | - | X |
| Diversity metrics  (S) | Fungal diversity | X | X | X |
|  | Bacterial diversity | X | X | X |
|  | Plant diversity | X | X | X |
| Plant response | Total plant biomass (aboveground) | X | X | X |
|  | Perennial plant biomass | X | X | X |
|  | Annual plant biomass | X | X | X |
| Decomposition  Variables* | CO₂ flux | X | X | X |
|  | Hydrophobicity | X | - | - |
|  | pH | - | - | X |
|  | Laccase | X | - | - |
|  | Mn-peroxidase | - | X | - |
| Soil Hydro-physical property* | Electrical conductivity (EC) | X | X | X |
|  | Water content (WC) | X | X | X |
| Soil Nutrients* | Total nitrogen (N) | X | X | X |
|  | Available phosphorus (P) | X | X | X |
|  | Potassium (K) | X | X | X |
|  | Iron (Fe) | X | X | X |
|  | Manganese (Mn) | X | X | X |
|  | Organic carbon (OC) | X | X | X |
| Bacterial phylum | Proteobacteria | X | X | X |
|  | Verrucomicrobiota | X | X | X |
|  | Acidobacteriota | X | X | X |
|  | Actinobacteriota | X | X | X |
| Fungal guild | Saprotrophs | X | X | X |
|  | Glomeromycota | X | X | X |
|  | Fungal parasites | X | X | X |
|  | Plant pathogens | X | X | X |
|  | Endophytes | X | X | X |

Table S2: Results of linear mixed-effects models including *Species* and *Zone* as fixed factors and ring identity as a random intercept to account for the nested sampling structure. F values and associated p values are based on Type III ANOVA with Satterthwaite approximation. The intraclass correlation coefficient (ICC) quantifies the proportion of total variance explained by between-ring variability. P values below α = 0.05 are reported in bold.

| Variable | Species | | Zone | | SpeciesxZone | | ICC |
| --- | --- | --- | --- | --- | --- | --- | --- |
|  | F value | P value | F value | P value | F value | P value |  |
| N° reads *M. oreades* | - | - | 34.9 | ***0.000*** | 34.6 | - | - |
| N° reads *A. vittadinii* | - | - | 2.7 | ***0.043*** | 2.7 | - | - |
| N° reads *C. collina* | - | - | 14.7 | ***0.000*** | 16.0 | - | - |
| S plants | 4.0 | ***0.047*** | 8.3 | ***0.002*** | 1.9 | 0.142 | 0.08 |
| H’ plants | 0.3 | 0.717 | 0.2 | 0.790 | 0.5 | 0.706 | 0.01 |
| Plant biomass (g/100 cm^2^) | 85.2 | ***0.000*** | 31.4 | ***0.000*** | 80.4 | ***0.000*** | 0.00 |
| N° reads fungi | 2.7 | 0.082 | 6.8 | ***0.003*** | 5.3 | ***0.002*** | 0.00 |
| ASV fungi | 9.5 | ***0.000*** | 43.9 | ***0.000*** | 4.4 | ***0.005*** | 0.00 |
| H'(log2) fungi | 6.4 | ***0.004*** | 7.4 | ***0.002*** | 8.9 | ***0.000*** | 0.00 |
| N° reads bacteria | 1.2 | 0.327 | 0.3 | 0.717 | 1.4 | 0.273 | 0.06 |
| ASV bacteria | 1.9 | 0.168 | 3.8 | ***0.031*** | 1.0 | 0.431 | 0.00 |
| H'(log2) bacteria | 1.7 | 0.222 | 3.9 | ***0.033*** | 1.3 | 0.313 | 0.04 |
| Hydrophobicity (s) | 4.4 | ***0.037*** | 4.7 | ***0.020*** | 7.3 | ***0.001*** | 0.01 |
| WHC (%) | 0.9 | 0.421 | 8.5 | ***0.002*** | 6.4 | ***0.001*** | 0.01 |
| pH | 3.8 | ***0.032*** | 137.5 | ***0.000*** | 13.5 | ***0.000*** | 0.00 |
| EC (µS/cm) | 66.1 | ***0.000*** | 405.5 | ***0.000*** | 148.9 | ***0.000*** | 0.00 |
| CO2 (mg/kg) | 144.7 | ***0.000*** | 347.0 | ***0.000*** | 251.9 | ***0.000*** | 0.02 |
| MB (mg/kg) | 175.4 | ***0.000*** | 321.9 | ***0.000*** | 81.3 | ***0.000*** | 0.00 |
| Mn Peroxidase (mmol) | 127.7 | ***0.000*** | 419.7 | ***0.000*** | 207.6 | ***0.000*** | 0.13 |
| Laccase (mmol) | 6.8 | ***0.011*** | 386.1 | ***0.000*** | 209.3 | ***0.000*** | 0.30 |
| OC (g/kg) | 4.8 | ***0.030*** | 183.3 | ***0.000*** | 7.7 | ***0.000*** | 0.19 |
| N (g/kg) | 0.8 | 0.493 | 136.6 | ***0.000*** | 1.3 | 0.303 | 0.22 |
| C/N | 4.3 | ***0.039*** | 7.9 | ***0.002*** | 3.3 | ***0.027*** | 0.02 |
| K (mg/kg) | 1.8 | 0.209 | 62.0 | ***0.000*** | 18.8 | ***0.000*** | 0.57 |
| Ca (mg/kg) | 1.9 | 0.192 | 56.0 | ***0.000*** | 2.1 | 0.112 | 0.15 |
| Na (mg/kg) | 119.3 | ***0.000*** | 48.8 | ***0.000*** | 47.5 | ***0.000*** | 0.01 |
| Mg (mg/kg) | 10.9 | ***0.000*** | 36.5 | ***0.000*** | 5.3 | ***0.002*** | 0.00 |
| P (mg/kg) | 5.6 | ***0.019*** | 108.0 | ***0.000*** | 15.1 | ***0.000*** | 0.10 |
| Fe (mg/kg) | 2.1 | 0.132 | 97.6 | ***0.000*** | 17.5 | ***0.000*** | 0.00 |
| Mn (mg/kg) | 8.8 | ***0.004*** | 173.5 | ***0.000*** | 24.0 | ***0.000*** | 0.10 |
| Zn (mg/kg) | 2.5 | 0.126 | 51.8 | ***0.000*** | 8.7 | ***0.000*** | 0.12 |
| Cu (mg/kg) | 7.9 | ***0.007*** | 8.6 | ***0.002*** | 31.6 | ***0.000*** | 0.15 |

Table S3: result of percent identity of sequences from fairy ring-forming fungi. Research made on NCBI the 10/11/2024

| **Morphology** | **ASV** | **Species NCBI** | **percent identity** | **Accession** |
| --- | --- | --- | --- | --- |
| *Marasmius oreades* | *Marasmius oreades* | *Marasmius oreades* | 100.00% | OQ282818 |
|  |  |  | 100.00% | MT908258 |
|  |  |  | 100.00% | OP784304 |
|  |  |  | 100.00% | OQ282804 |
|  |  |  | 100.00% | CP097452 |
|  |  |  | 100.00% | CP097441 |
|  |  |  | 100.00% | OP470638 |
|  |  |  | 100.00% | OP455769 |
|  |  |  | 100.00% | ON166656 |
|  |  |  | 100.00% | JN943604 |
| *Amanita vittadinii* | *Amanitaceae_incertae_sedis* | *Amanita vittadinii* | 100.00% | MH603603.1 |
|  |  |  | 100.00% | MK512062.1 |
|  |  |  | 100.00% | ON782460.1 |
|  |  |  | 100.00% | OP271579.1 |
|  |  |  | 100.00% | MH508651.1 |
|  |  |  | 99.20% | OP271577.1 |
|  |  |  | 99.72% | OP850821.1 |
| *Clitocybe collina* | *Clitopilus sp.* | *Clitopilus sp.* | 95.64% | GU083222.1 |
|  |  |  | 95.64% | OP751538.1 |
|  |  |  | 95.07% | FJ770402.1 |
|  |  |  | 94.22% | OW846751.1 |
|  |  |  | 94.19% | KT581706.1 |
|  |  |  | 94.19% | EU490059.1 |
|  |  |  | 94.19% | MK217426.1 |

Table S4: Result of Permutation analysis of variance (PERMANOVA) on changes in fungal community at ASV level for FRs position and species/type. Pseudo-F describing effect size are reported; significance threshold fixed for values of p below 0.05.

|  | **df** | **SS** | **MS** | **Pseudo-F** | **P(perm)** |
| --- | --- | --- | --- | --- | --- |
| Zone | 2 | 13167 | 6583.6 | 6.82 | 0.001 |
| Species | 2 | 9214 | 4607 | 4.77 | 0.001 |
| Zone vs Species | 4 | 12596 | 3149 | 3.26 | 0.001 |
| Residuals | 35 | 33744 | 964.11 |  |  |
| Total | 43 | 67692 |  |  |  |

Table S5: Result of Permutation analysis of variance (PERMANOVA) on pairwise comparisons in fungal community at ASV level of FRs zones for each FR fungus: of *M. oreades* (type 1). *A. vittadinii* (type 2). *C. collina* (type 3). T values describing effect size are reported; significance threshold fixed for values of p below 0.05.

|  | ***M. oreades*** | | ***A. vittadinii*** | | ***C. collina*** | |
| --- | --- | --- | --- | --- | --- | --- |
|  | **t** | **P(perm)** | **t** | **t** | **P(perm)** | **t** |
| IN vs FF | 2.58 | *0.009* | 1.35 | *0.026* | 1.81 | *0.038* |
| IN vs OUT | 1.70 | *0.014* | 1.98 | *0.004* | 2.18 | *0.006* |
| FF vs OUT | 3.12 | *0.006* | 1.87 | *0.012* | 2.24 | *0.005* |

Table S6: Result of Permutation analysis of variance (PERMANOVA) on changes in bacterial community at ASV level for FRs position and species/type. Pseudo-F describing effect size are reported; significance threshold fixed for values of p below 0.05.

|  | **df** | **SS** | **MS** | **Pseudo-F** | **P(perm)** |
| --- | --- | --- | --- | --- | --- |
| Zone | 2 | 3005.9 | 1503 | 2.51 | 0.001 |
| Species | 2 | 4212.1 | 2106.1 | 3.51 | 0.001 |
| Zone vs Species | 4 | 5222.1 | 1305.5 | 2.18 | 0.001 |
| Residuals | 36 | 21543 | 598.41 |  |  |
| Total | 44 | 33983 |  |  |  |

Table S7: Result of Permutation analysis of variance (PERMANOVA) on pairwise comparisons in bacterial community at ASV level of FRs zones for each FR fungus: of *M. oreades* (type 1). *A. vittadinii* (type 2). *C. collina* (type 3). T values describing effect size are reported; significance threshold fixed for values of p below 0.05.

|  | ***M. oreades*** | | ***A. vittadinii*** | | ***C. collina*** | |
| --- | --- | --- | --- | --- | --- | --- |
|  | **t** | **P(perm)** | **t** | **t** | **P(perm)** | **t** |
| IN vs FF | 1.53 | 0.008 | 1.56 | 0.011 | 1.69 | 0.031 |
| IN vs OUT | 1.63 | 0.008 | 1.41 | 0.061 | 1.56 | 0.057 |
| FF vs OUT | 1.19 | 0.168 | 1.28 | 0.102 | 1.74 | 0.018 |

Table S8**.** Composite reliability (ρₐ) and Average Variance Extracted (AVE) for latent constructs included in the species-specific PLS-SEM models. Values are reported separately for *Marasmius oreades*, *Amanita vittadinii*, and *Clitocybe collina* fairy rings. Composite reliability ρₐ was used as the primary reliability criterion. as it provides a conservative and robust estimate in PLS-SEM. particularly for models with a limited number of indicators.

| **Latent construct** | ***M. oreades*** | | ***A. vittadinii*** | | ***C. collina*** | |
| --- | --- | --- | --- | --- | --- | --- |
|  | *ρₐ* | *AVE* | *ρₐ* | *AVE* | *ρₐ* | *AVE* |
| Decomposition | 0.987 | 0.972 | 0.959 | 0.959 | 0.737 | 0.765 |
| Hydro-physical properties | 0.924 | 0.929 | 0.798 | 0.712 | 0.940 | 0.906 |
| Nutrients | 0.952 | 0.709 | 0.974 | 0.897 | 0.955 | 0.806 |

Table S9**.** Global model fit indices based on saturated models for the species-specific PLS-SEM analyses. Only saturated model values are reported. as recommended for comparative diagnostic purposes in PLS-SEM.

| **Fairy ring type** | **SRMR (saturated)** | **d_ULS (saturated)** |
| --- | --- | --- |
| *M. oreades* | 0.162 | 14.747 |
| *A. vittadinii* | 0.080 | 2.080 |
| *C. collina* | 0.093 | 3.016 |

**Notes.** SRMR. standardised root mean square residual; d_ULS. squared Euclidean distance. Other global fit indices (Chi-square. NFI. d_G) are not reported. as they are not applicable or not informative in the context of PLS-SEM.
